# Gendered mediating role of substance use in the path from internet addiction or gambling to active violence in youth in the region of Mahdia, Tunisia

**DOI:** 10.3389/fpsyt.2025.1627108

**Published:** 2025-11-03

**Authors:** Imen Mlouki, Safa Hssan, Emna Hariz, Aya Ajmi Blout, Ahlem Silini, Ahmed Moustafa, Mohamed Hedi Ben Cheikh, Sana El Mhamdi

**Affiliations:** ^1^ Department of Preventive and Community Medicine, Mahdia University Hospital, Mahdia, Tunisia; ^2^ Research Laboratory “Epidemiology Applied to Maternal and Child Health” 12SP17, Department of Preventive and Community Medicine, Faculty of Medicine of Monastir, University of Monastir, Monastir, Tunisia; ^3^ Department of Human Anatomy and Physiology, the Faculty of Health Sciences, University of Johannesburg, Johannesburg, South Africa; ^4^ Centre for Data Analytics & School of Psychology, Bond University, Gold Coast, QLD, Australia; ^5^ Department of Pharmacy, Mahdia University Hospital, Mahdia, Tunisia

**Keywords:** internet addiction disorder, gambling, substance-related disorders, violence, adolescents, tunisia

## Abstract

**Introduction:**

The literature shows a gap regarding mechanisms and mediators explaining the path from IA and gambling to violence. Our study aimed to investigate the gender-specific difference in the path linking IA and gambling to active violence mediated by substance use among schooled adolescents in the region of Mahdia (Tunisia).

**Methods:**

We performed a cross-sectional survey among schooled youth in the region of Mahdia in April 2023. We conducted a mediation analysis using in SPSS the PROCESS macro to examine the relationship between X (IA/Gambling), the mediator M (Substance use), and Y (Active violence). Causal interpretations cannot be definitively established. We used validated Arabic versions of the Internet Addiction Test, the South Oaks Gambling Screen Test, the CDC Health Appraisal section about substance use and the Hospital Anxiety and Depression Scale. We also probed violence perpetration. Data analyses were performed using SPSS version 20.

**Results:**

A total of 1594 students completed the questionnaire with a mean age of 17.75 ± 1.34 years. IA was noted in 52.3% of the participants. Gambling was more prevalent among males (12.1% vs 0.9%, p<0.001). Substance use was noted in 18.4% of participants (41.3% males vs 9.5% females, p<0.001). Violence perpetration was reported by 35.3% of the participants (59.9% males vs 25.8% females, p<0.001).

After accounting for anxiety and depression, substance use mediated the link between IA and active violence (mediation%= 15.7, p< 0.001) with a higher effect in males (35.8% vs 10.5%). The mediation between gambling and violence via substance use was also significant (28.2%, p < 0.001). This effect was found only among males (27.7%).

**Conclusion:**

Implementing preventive strategies and focusing on addictive behaviors such as IA, gambling and substance use is urgently needed to prevent violence perpetration among youth. However, based on our results, the cross-sectional design limits causal interpretations.

## Introduction

Adolescence is a critical maturational period manifested by significant physical, cognitive, and social-emotional transformations (1). During this time, youths are particularly susceptible to problematic and risky behaviors, such as addictive behaviors and violent actions. Youth violence is a significant global concern. In fact, the World Health Organization (WHO) estimates that 37% of total global homicides are among adolescents and the majority of them are males (2). According to the Global School-based Student Health Survey (GSHS) (3), one in ten students experienced multiple physical fights in 2019. The highest prevalence was recognized in North Africa (46.3%) and the lowest in Central America (25.6%). Across all GSHS countries, it is important to note the gender differences, with 11.4% of males and 4.7% of females experiencing a high level of active violence (3). In the United States (US), violence among youth costs through homicides and nonfatal physical assault-related injuries $122 billion annually in 2020. In Tunisia, an increase in violent incidents among schooled adolescents was reported as 3,398 in 2021 to 3,650 in 2023 (4). For example, the prevalence of active violence in the “Zgana” primary school in Mahdia was 19% in 2021 (5) and in middle schools of Sfax was 73.6% (6). According to gender, males exhibited higher levels of violent behaviors compared to females (6).

Internet addiction (IA) and gambling are also common risky behaviors in adolescents. More specifically, high percentages of IA among youth were reported in North Africa between 2013 and 2021(44.6%) with a rate of 43.9% explicitly observed in Tunisia (7). According to gender, some surveys reported that males were more addicted to internet. In contrast, other studies showed that females were more addicted than males (7, 8). Regarding gambling, the global prevalence among adolescents was 17.9% (9). In 2021, this rate varied between countries reaching 3.6% across European countries (10) and 32.8% in Tunisia (11). According to literature (9–11), more males were engaged in gambling than girls.

Research has consistently demonstrated that both overuse of the internet and gambling problem lead to increased violent behaviors especially among adolescents (12, 13) In fact, a 2023 review analysis in the United Kingdom (UK) (14) revealed that suicide rates were 15 times higher among youth suffering from gambling addiction compared to the general population. Furthermore, the costs of criminal activities linked to gambling were estimated at £167.3 million (14). Concurrently, other research has documented the alarming relationship between pathological internet use and heightened risks of premature mortality. Indeed, youth with IA are often exposed to violent content such as violent games, which desensitizes them to aggression (15). This, combined with insomnia and social isolation, leads to increased frustration and irritability. Similarly, neuroimaging studies have reported that both IA and gambling are linked to functional changes in brain regions involved in executive control and emotional regulation. As a result, adopting these risky behaviors is related to increased impulsivity, poor decision-making and maladaptive coping strategies including aggressive behavior (16–20).

Substance use also remains a significant global public health concern, particularly among young people. Cannabis remains the most popular illicit substance use among youth, with approximately 4.7% of adolescents in 2018 (21). Additionally, tobacco use affects a sizeable proportion of youths globally, with at least one in ten adolescents aged 13–15 using tobacco (21). Over 50% of youth aged 15 years experimented with high levels of alcohol consumption, and one in five use e-cigarettes in Europe, Central Asia, and North America. In the USA, it is estimated that at least one in eight adolescents has abused an illicit substance, leading to thousands of overdose deaths every year. In Tunisia, alarming rates were nationally reported among students being 30.7% for tobacco, 8% for alcohol, and 7.9% for cannabis in 2021 (11). As for gender, Tunisian male adolescents were more engaged in substance use than girls (11).It was reported that substance use was associated with aggressive behavior by intensifying mood disorders and impairment of self-regulation and decision-making (22, 23).

Despite the existing evidence on the links between those risky behaviors and youth violence, there is a notable gap in the literature regarding the specific mechanisms explaining the path from gambling or IA to active violent behaviors, particularly among Tunisian adolescents. Investigating the mediating role of substance use according to gender in this pathway could provide valuable insights for developing targeted prevention and specific intervention strategies.

Thus, the aim of the current study was to investigate the gender-specific difference in the path linking IA and gambling to active violence mediated by substance use among schooled adolescents in the region of Mahdia.

## Methodology

### Study design and participants

The present research employed a cross-sectional design in April 2023 among schooled youth in the region of Mahdia (Tunisia). We aimed for representativeness by selecting adolescents from all public secondary schools in Mahdia city.

Participant selection was done according to the cluster sampling method. In fact, from each educational level (four levels), one class was randomly chosen. Then, all students in the selected classes who met the inclusion criteria and agreed to participate were included. The inclusion criteria encompassed individuals with the necessary cognitive ability to read, understand and respond to the questionnaire correctly. Informed consent from either the participants themselves or their legal guardians was also required. We excluded incomplete questionnaires from the final analysis.

To ensure an adequate sample size for representativeness, the required minimal number of students (n) was 657 determined based on a 19% prevalence (p) of active violence among Tunisian teenagers (5),a 0.05 type one error (α) and an accuracy (i) of 3%. It was calculated using the formula n= (p x (1 – p) x (Zα/2)²)/i².

### Data collection procedure

Participants were given self-report questionnaires in Arabic during school hours in a suitable and private setting. Using anonymous tools in native language and conducting the survey in a familiar school environment were strategies to minimize response bias, while also ensuring confidentiality. In fact, only the research team was present during data collection in order to clarify any doubts and to encourage honest and accurate responses.

### Measures and tools

#### Internet addiction

IA was assessed using the validated Arabic version of the IA Test (IAT) (24), a 20-item measure that captures individual levels of problematic internet use, including preoccupation, withdrawal symptoms, loss of control, and negative consequences. The scores on the IAT range from zero to 100. A total score below 39 indicated a normal level of internet use, while a score of 40 or higher indicated problematic use, reflecting moderate to severe internet dependence (24).

#### Gambling behavior

Gambling behavior was measured using the South Oaks Gambling Screen Revised for Adolescents (SOGS-RA) (25).We used the Arabic version employed in the national MEDSPAD study among youth in 2021 (11). The SOGS-RA includes 12 items that assess gambling behaviors and experiences over the past 12 months at least once. Total scores can range from zero to 12, with higher scores indicating more severe gambling problems (11). Standardized cut-off scores are used to classify respondents into categories of problem gambling severity: A score equal to zero or one indicates no problem gambling. A score of two or three indicates at risk. A score equal to or greater than four indicates problem gambling.

#### Substance use

We used the Arabic version of the CDC Health Appraisal section about substance use (26) to assess substance use, including tobacco, e-cigarette smoking, alcohol consumption, and illicit drug use (cannabis). Participants were scored based on their reported use of different substances tobacco, alcohol, electronic cigarettes, and cannabis: from zero (no reported use of any substance) to four (reported use of all substance types including traditional cigarette, e-cigarette, alcohol, and cannabis).

#### Youth active violence

To assess active violence among students, we employed a scoring system that categorized individuals based on the severity of aggressive behavior. Zero when no active violence was reported, score one if the student reported active violent behavior and score two for those being violent with the use of weapons or causing injuries. We also investigated the place of violence perpetration (Home, school, or street).

#### Anxiety/depression

We utilized the validated Arabic version of the Hospital Anxiety and Depression Scale (HADS) to screen for anxiety and depression (27). The HADS consists of two subscales for anxiety and depression, each with seven items rated on a 4-point Likert scale (0-3). Participants were identified as screening positive for anxiety or depression if their total anxiety or depression score exceeded the cut-off of eight points.

### Statistical analysis

Data analyses were performed using SPSS version 20. Descriptive statistics, including absolute and relative frequencies for qualitative variables, and means and standard deviations for quantitative variables, were calculated. Chi-square and student t-tests were used to compare percentages and means, respectively, for assessing differences between groups. A significance level (p-value) of <0.05 was used to determine if the results were statistically significant.

**Figure 1:**
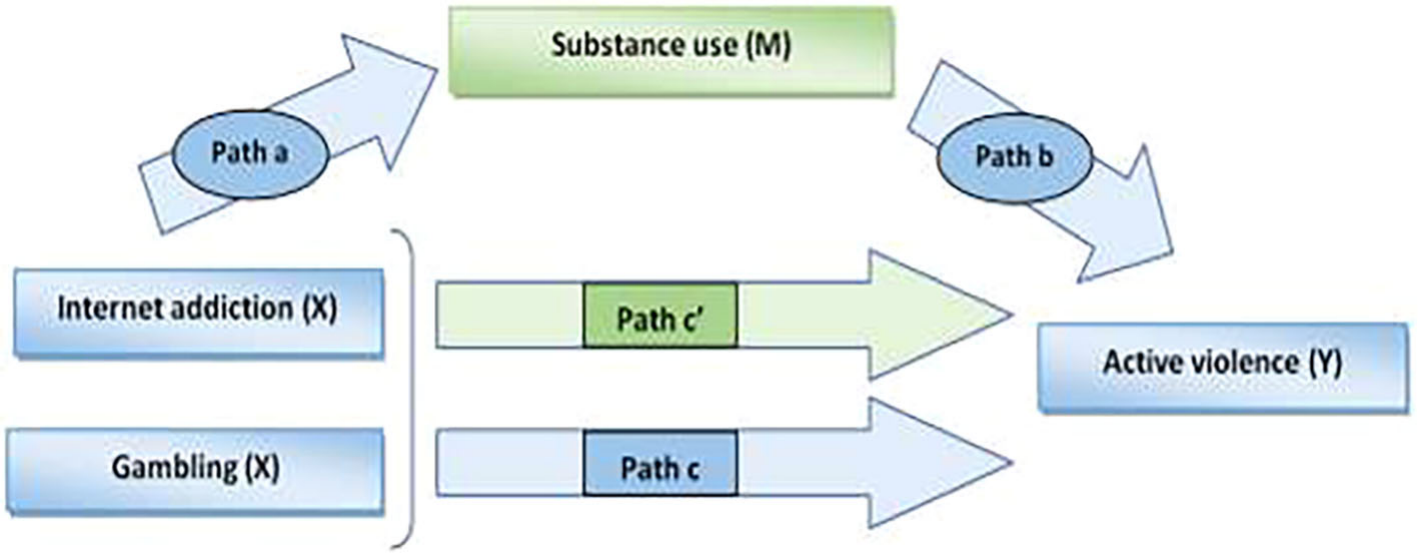
Diagram of the mediation pathway model: IA/Gambling, Substance use and active youth violence.

We conducted a mediation analysis using in SPSS the PROCESS macro developed by Andrew F. Hayes (28)to examine the relationship between the independent variable X (IA/Gambling), the mediator M (Substance use), and the dependent variable Y (Active violence). The mediation analysis was also adjusted for gender and anxiety (**Figure 1**). The Sobel test (29)was used to assess and verify the indirect effect. In fact, the inclusion of the mediator should lead to a total or partial reduction (path c’) of the effect between the independent variable and the dependent variable (path c).

For mediation to be conducted, all three pathways (a, b, and c) must be significant. We used the correlation test to verify these links.

### Ethical considerations

The study protocol was approved by the Ethics Committee at the University Hospital of Mahdia (Tunisia) under Approval Number P01 M.P.C-2020.

Prior to data collection, permissions were sought from the headmasters of the schools involved in the study. Adolescents and their parents were provided with a clear explanation of the use of test results for research purposes, and they were given the freedom to decline participation if they so wished. The questionnaire was administered anonymously and self-administered by the participants. Trained doctors supervised the data collection process, ensuring that no teachers or administrative staffs were present during the administration. Additionally, a separate trained member was responsible for data entry.

## Results

A total of 1594 students aged between 15 and 22 returned completed questionnaires with a response of 86.6%. The mean age of participants was 17.75 ± 1.34. Among them, 72% were identified as female.

### Health risk behaviors among schooled adolescents by gender, Mahdia 2023

#### Internet addiction and gambling disorder

Our findings revealed an IA rate of 52.3% among students. According to gender, there is no significant difference in IA between females and males (53.4% vs 49.7%, p=0.18). We found that 4% adolescents were identified as active gamblers. Among them, 42.1% were at risk and 57.8% had gambling problem. Males showed higher rates of gambling compared to females (12.1% vs 0.9%, p<0.001).

#### Substance use and active violence

As shown in **Table 1**, substance use was reported in 18.4% of cases. According to gender, males were significantly more engaged. Engagement in active violence was reported by 35.3% of the sample. The average age at which participants reported their initial violent act was 11.83 ± 3.02 years. Using weapons or causing injuries were reported by 51.5% of students who were engaged in violent incidents.

**Table 1:** Gender-based patterns in adolescent substance use and active violent behaviors, Mahdia 2023.

Substance use and active violent behaviors	Total n(%)	Male n(%)	Female n(%)	P value
Tobacco use (N=1593)	80(5)	69(**15.5**)	11(1)	**<0.001**
E-cigarette use (N=1591)	246(15.5)	150(**33.7**)	96(8.4)	**<0.001**
Alcohol use (N=1591)	70(4.4)	56(**12.6**)	14(1.2)	**<0.001**
Cannabis use (N=1592)	50(3.1)	43(**9.6**)	7(0.6)	**<0.001**
Substance use	292 (18.4)	183(**41.3**)	109(9.5)	**<0.001**
The mean age of violence engagement ±SD	11.83±3.02	**11.34± 2.96**	12.22 ±3.02	**p=0.035**
Active violence, N=1594				**<0.001**
Yes without weapons or injuries	273(17.1)	91 (**20.4**)	182 (15.9)	
Using weapons or causing injuries	290(18.2)	176 (**39.5**)	114 (9.9)	
Setting, N=748				**<0.001**
Home	219(29.12)	52 (17.68)	167(**36.46**)	
School	350(46.54)	140 (**47.61**)	210 (45.85)	
Street	442(58.77)	222 **(75.51**)	220 (48.03)	

Statistically significant results (p < 0.05) are shown in bold.

Statistical analyses by gender revealed that 59.9% of males reported being violent (versus 25.8% of females, p<0.001). Finally, male youth were significantly more likely to be violent on the street, while female youth were more violent at home (**Table 1**).

### Anxiety and depression among schooled adolescents by gender, Mahdia 2023

The overall prevalence of anxiety and depression were 44.9% and 62% respectively. When comparing rates of depression stratified by gender, we found statistically a significant gender gap (68.6% among females compared to 44.6% among male youth, p<0.001).

### Correlation pattern linking behavioral addictions, substance use, and violent tendencies among students

Statistically significant correlations (p<0.001) were found between all the variables examined in this study. More specifically, gambling behavior score was found to have a positive correlation with both substance use score (r=0.29) and active violence score (r=0.21). Similarly, higher levels of IA score were associated with increased score of substance use (r=0.11) and active violent behaviors (r=0.13). Finally, a positive correlation between substance use and active violent behavior scores was found (r=0.31, p<0.001).

**Table 2** presents these associations based on binary variables categorized by gender.

**Table 2:** Associations between adolescent IA, gambling, substance use and violent behaviors according to gender, Mahdia, 2023.

Active violence	Male n(%)		p	Female n(%)		p	pTotal
	yes	no		yes	no		
Internet addiction			**0.06**			**<0.001**	**<0.001**
N=1557							
YES	139(64.7)	76(35.3)		193(32.2)	407(67.8)		
No	122(56)	96(44)		98(18.7)	426(81.3)		
Gambling			**0.01**			**<0.013**	**<0.001**
N=1593							
Yes	41(75,9)	13(24,1)		6(60)	4(40)		
No	226(57.7)	166(42.3)		290(25.5)	847(74.5)		
Substance use			**<0.001**			**<0.001**	**<0.001**
N=1589							
Yes	128(69.9)	55(30.1)		54(49.5)	55(50.5)		
No	137(52.7)	123(47.3)		241(23.2)	796(76.8)		

Statistically significant results (p < 0.05) are shown in bold.

### The path from addictive behaviors to being violent among adolescents: Mediation analysis by substance use according to gender

**Figure 2:**
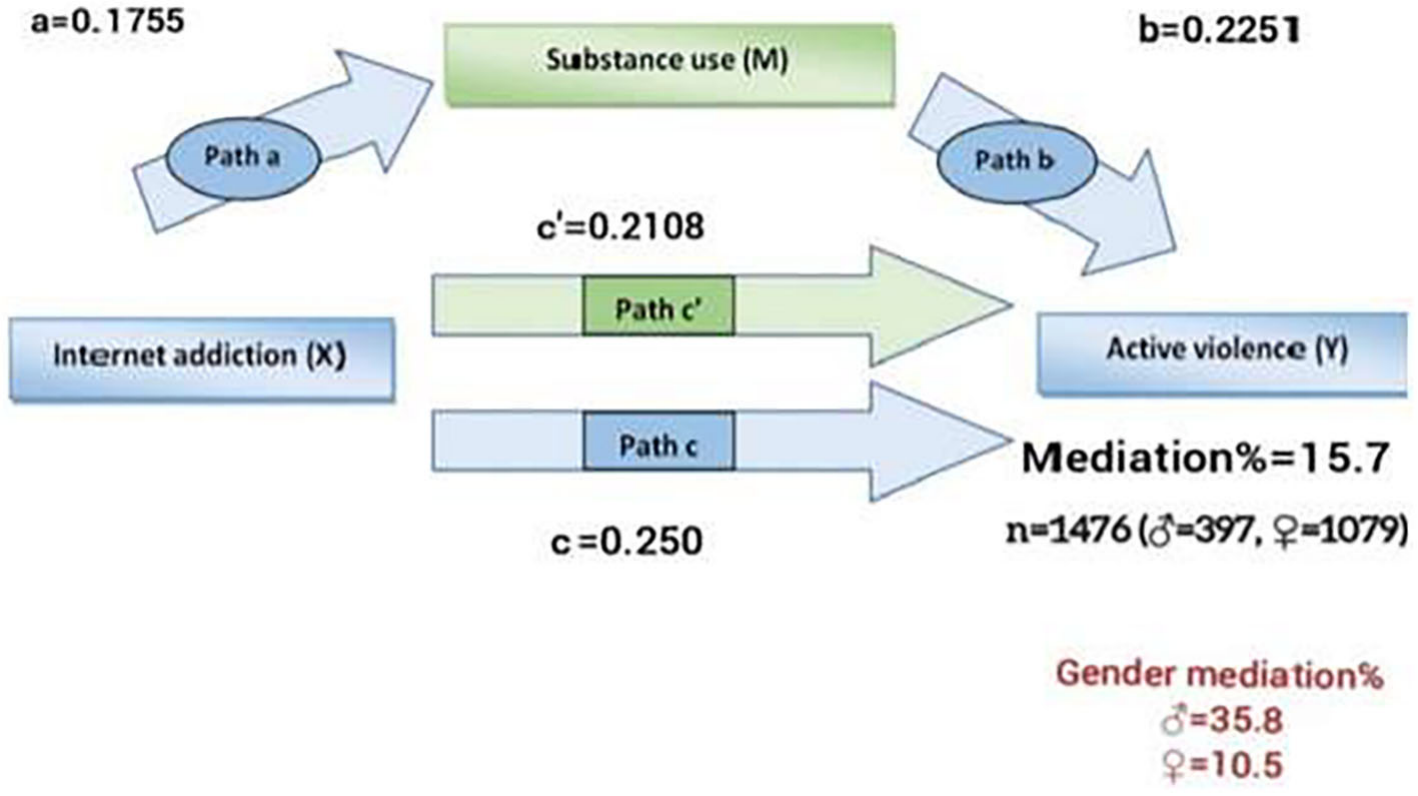
Gendered mediating effect between IA, substance use as mediator, and youth active violence with anxiety and depression as covariates (p< 0.001).

**Figure 2** illustrates the results of the mediation analysis regarding IA as the independent variable. After adjusting for anxiety and depression, the indirect effect of IA on youth active violence through substance use was statistically significant (mediation%= 15.7, p < 0.001). Gender also emerged as a significant predictor in the model. Specifically, the male gender was associated with a higher rate of mediation compared to females (35.8% vs 10.5%) (**Figure 2**).

**Figure 3:**
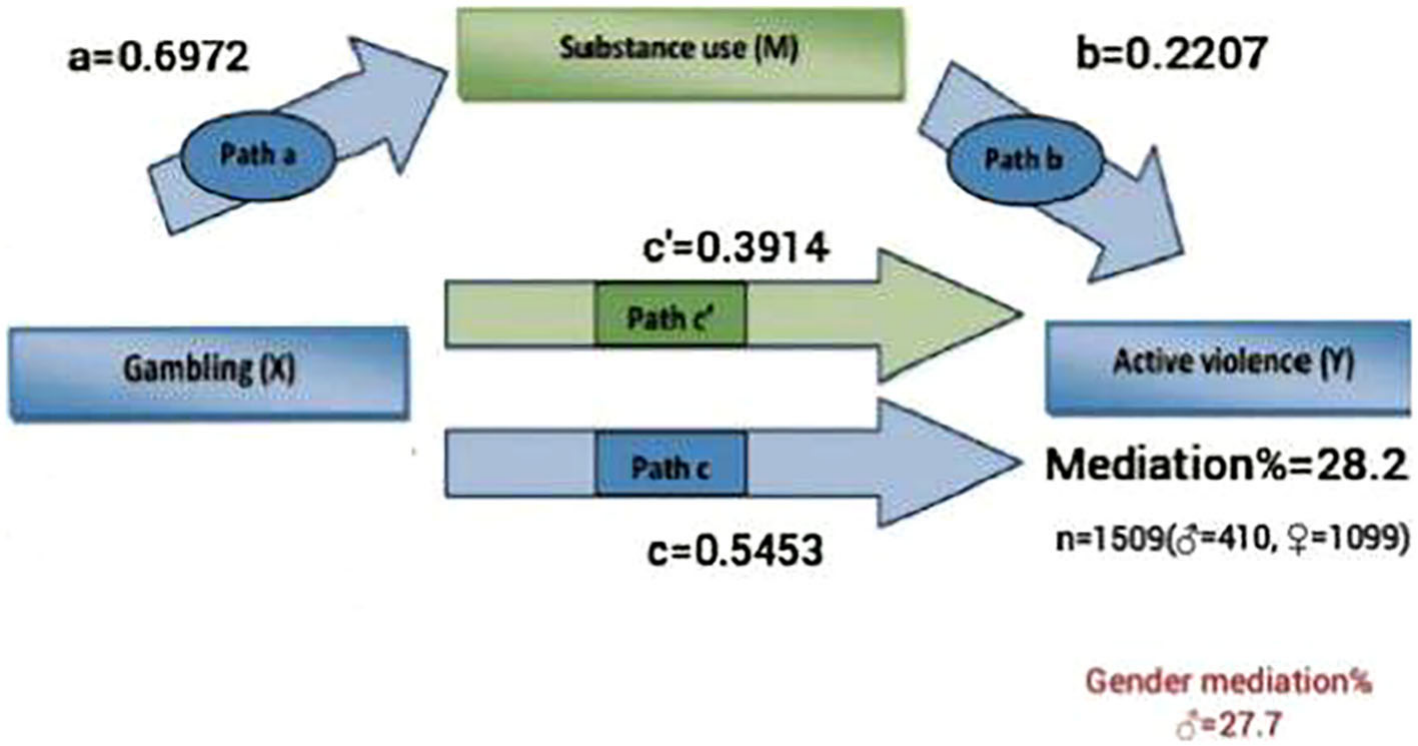
Mediation model by gender examining the indirect effect of adolescent gambling behaviors on active violence through substance use adjusted for anxiety and depression (p<0.001).

As shown in **Figure 3**, the total mediation effect of gambling on active violence via substance use was significant (mediation= 28.2%, p <0.001) when adjusting the mediation model for anxiety and depression. According to gender, the mediation effect was significant and reached 27.7% among males and no mediation effect was found for girls (**Figure 3**).

## Discussion

Interestingly, our research revealed the mediating role of substance use in the connections between IA, gambling, and active violence among a sample of schooled adolescents in the region of Mahdia. In fact, we found that substance use significantly mediated the link between IA and active violence at 15.7% (p <0.001), with a striking effect in males (35.8% vs. 10.5% for females). The impact is similarly profound for gambling, with a mediation effect of 28.2% (p <0.001), observed exclusively in males (27.7%). The current study fills a critical gap in the literature globally, but especially North African and the Middle East.

A large proportion of our sample was females (72%). This could be due to the greater enrollment of girls in schools within Tunisian expectations that promote girls education in contrast boys may exhibit higher rates of absenteeism. Our research reveals a prevalence of 52.3% of IA among adolescents with no significant gender difference. This rate is notably higher than the reported rates in the region of Turkey (4.8%), Qatar (29.64%) and China (12.8%) (30). In Tunisia, it is worth noting that the prevalence of IA among schooled youth in the same region (Mahdia) was 50% in 2020 (31). This increase can be attributed to economic challenges, the impact of the COVID-19 pandemic and a lack of recreational activities in Tunisia which drive individuals to spend more time online as a form of escapism. According to prior studies, several related factors were reported including childhood trauma, dysfunctional family atmosphere, low self-esteem, peer influence and academic pressure as key drivers of IA (30, 32, 33). The absence of gender difference in IA in our study could be explained by the widespread availability of the internet as well as Smartphone among male and female adolescents. While motivations vary, girls seek social connections and males are more inclined toward gaming. Our research emphasizes the link between IA and substance use (path a) among youth, supporting findings from other studies. Internet platforms expose young people to substance use, shaped by their peers and influencers, which makes them perceive this behavior as acceptable (34–36).In addition, our findings showed an association of IA with active violence (path b) among adolescents which aligns with other studies. Excessive internet use deprives adolescents of the opportunity to develop social skills and participate in positive activities. When engaging in real life, they may struggle to express their emotions and socialize, contributing to irritability and violent tendencies (12, 15, 37–39).

Regarding gambling, we found that gambling problems were screened among 4% of youth with a notable gender disparity (12.1% in males and 0.9% in females). This aligns closely with the rates of 4.7% in Southern Spain and 6.4% in Italy (40, 41). However, the prevalence was significantly higher in the Tunisian national study reaching 30.1% in 2021 (11).This difference may be attributed to limited access to gambling settings for residents of Mahdia compared to more urban or diverse regions in Tunisia. Many young people in Tunisia gamble to escape financial difficulties, motivated by a desire for excitement and sensation-seeking. This behavior influenced by strong peer and parental pressures, results in higher participation rates among adolescents. Additionally, the lack of awareness and educational programs about the risks associated with gambling further exacerbates the problem (42, 43).Gambling can lead to numerous negative consequences including financial difficulties, mental health problems such as anxiety and depression. It also adversely affects academic performance and interpersonal relationships. Furthermore, our study, along with other research, showed that gambling is associated with heightened risks of active violence (path b) and substance use (path a) (42, 44–46). Many gambling environments involve the consumption of substance use combined with high competition emotions can lead so to increased aggression.

Concerning violent behaviors among youth, this survey found a worrying prevalence with 35.3% of students reporting actively engaging in violent acts. This is notably higher than the rates reported among adolescents in Western countries including Europe, Central Asia and Canada (10%), in Kuwait (25.2%) and Sub-Saharan Africa (29.2%). In contrast, the rates in India (46.7%), Ghana (38.2%) and Colombia (57.52%) are above our overall finding (47–52). This figure is particularly striking when considering the significant gender disparity, where 59.9% of males reported violent behavior (vs 25.8% of females). A possible explanation for this is the cultural norm often promotes aggression as a sign of male strength, contributing to higher rates of violent behavior among boys. In contrast, females may act aggressively using indirect ways through relational means, such as social manipulation or exclusion, rather than through active violence. Another key finding is the average age of the first violent act, reported at 11.83 years. Active violent behavior tends to emerge more frequently in early adolescence than in late adolescence. This early development of violent behavior is alarming, as it can normalize aggression and increase the risk of ongoing violence in later adolescence and adulthood. Streets (58.7%) and schools (46.5%) were the most common locations for violent incidents in our study, indicating a need for effective conflict resolution strategies in these areas. Moreover, the use of weapons or causing injuries was reported by 51.5% of those who engaged in violence. This statistic not only reflects the severity of the violent acts but also raises concerns about access to weapons and the normalization of such behavior among youth who may perceive it as a means to feel secure.

Substance use was common in our sample (18.4%) with a notable gender disparity (41.3% of males compared to 9.5% of females). When comparing these rates internationally, the substance use prevalence in our sample is significantly higher than in Ghana (12.3%), Tanzania (12.8%) and Germany (11.2%), but remains lower than observed in Northwest Ethiopia (52.5%) and India (32.8%) (53–57). In our study, the specific rates of substance use included tobacco (**5%**), e-cigarettes (15.5%), alcohol (4.4%), and cannabis (3.1%). In contrast, a 2020 Tunisian study in the same region (Mahdia) reported higher rates: 12.4% for cigarette smoking, 16.4% for e-cigarette use, 9.9% for alcohol consumption, and 6.8% for cannabis consumption (58). These varying rates can be linked to difference in sample size, types of substances being studied, along with sociocultural and economic factors. Additionally, the accessibility of substances in Tunisia plays a significant role in influencing patterns of substance use.

Concerning the mediation models, our study contributes to the growing body of literature. Unfortunately, no studies in the literature have explored gendered mediation models between IA or gambling and active violence through substance use. This makes difficult to compare our results with those of other studies. While prior research has explored connections, our findings showed that substance use mediated the path from IA and gambling to aggressive behavior in adolescents. In fact, IA and gambling can create environments where adolescents encounter heightened emotional states, such as stress and frustration promoting them to seek coping mechanisms (16, 38, 46, 59, 60). An increase dopamine activity in the dorsal striatum, stimulated by both IA and gambling, enhances reward-seeking behavior (76). This heightened sensitivity to rewards can drive adolescents to pursue substance use in search of similar pleasurable effects. Neuroimaging studies show reduced activity in the ventral striatum and prefrontal cortex, which impairs decision-making and impulse control (17–20, 60, 61). As a result, once substance use is established, it can further impair judgment and decrease impulse control, significantly increasing the risk of aggressive behavior (22, 23, 62). This perspective underscores the necessity of addressing substance use in the context of the neurochemical and psychological challenges that adolescents encounter with IA or gambling. Doing so is essential for understanding and reducing the risks of violence linked to these behaviors. Added to that, our research showed that considering gender differences is crucial in understanding this path from addictive behaviors to active violence. Gender differences found, align with other studies, in gambling (9–11, 40, 44, 63), substance use (11, 53–56, 58), active violence (3, 6, 48–50), and the mediation effects involved supporting existing literature that shows males are more likely to take risk behaviors. Furthermore, cultural norms encourage males to risky behaviors and discourage them from expressing their emotions, which increases their vulnerability to mental health issues (64).When considering gender in mediation, we found that substance use significantly mediated the relationship between IA and active violence, with a notable effect in males. However, no mediation was observed in gambling for females, as the mediation effect was exclusively present in males. This can be explained by the lower prevalence of gambling among females (0.9%), which likely limits the opportunity to observe a mediation effect between gambling and active violence in this group.

The present research has several limitations that should be considered when interpreting the findings. First, the reliance on self-report questionnaires may introduce response bias, as participants might underreport or exaggerate certain behaviors. Additionally, this study focused exclusively on schooled adolescents, which limits the generalizability of the findings because out-of-school youth may exhibit different patterns of risky behaviors. Furthermore, the research did not investigate potential protective factors that could mitigate the risks associated with the behaviors studied. While anxiety and depression were controlled for, other confounding variables such as peer influence, family dynamics, and socioeconomic status were not assessed and may impact the relationships examined.

Furthermore, we recognize that mediation analysis based on cross-sectional, self-reported data cannot establish strong causal interpretations. The findings should be viewed as correlational, and assumptions about the timing and direction of effects should be interpreted with caution. Future research may be able to address this limitation by using a prospective longitudinal approach.

However, the rise of digital technologies and risky behaviors makes our data highly needed today, especially among vulnerable populations. This research increases awareness of healthcare providers, policymakers, educators, and family members. Additionally, it fills a gap in the literature by linking addictive behaviors to active violence among adolescents. Gender-specific analysis enriches the research, as it paves the way for other studies to deeply understand this association. Using a cross-sectional design with validated questionnaires adds to the credibility of our findings. Furthermore, a total of 1594 strengthens the statistical analyses and the generalizability of our results. The use of the mediation analysis makes a specific and strong methodological framework.

## Conclusion

This study has established that substance use serves as a significant mediator in the relationship between IA, gambling disorders and youth active violence, particularly highlighting gender-specific differences with a striking effect in males. These findings highlight the need for prevention and intervention strategies that address the distinct ways in which risky behaviors show up in male and female youth. Effective treatment approaches should not only focus on internet use and gambling but also consider substance use therapeutic interventions. Targeting substance use may be especially effective in disrupting the pathway to violence among male youth. By adopting a multidimensional approach, youths can manage challenges associated with these risky behaviors, ultimately reducing the incidence of violence in this vulnerable population.

## Data Availability

The original contributions presented in the study are included in the article/supplementary material. Further inquiries can be directed to the corresponding author.
